# Early Treatment with Anti-VLA-4 mAb Can Prevent the Infiltration and/or Development of Pathogenic CD11b^+^CD4^+^ T Cells in the CNS during Progressive EAE

**DOI:** 10.1371/journal.pone.0099068

**Published:** 2014-06-04

**Authors:** John E. Mindur, Naoko Ito, Suhayl Dhib-Jalbut, Kouichi Ito

**Affiliations:** Department of Neurology, Rutgers-Robert Wood Johnson Medical School, Piscataway, New Jersey, United States of America; Kyushu University, Japan

## Abstract

Natalizumab is a humanized monoclonal antibody against the leukocyte adhesion molecule very late antigen (VLA)-4, and is currently an approved therapy for patients with relapsing-remitting multiple sclerosis (RRMS). However, it is unknown whether natalizumab is beneficial for progressive forms of MS. Therefore, we assessed the effects of anti-VLA-4 monoclonal antibody (mAb) therapy in a progressive experimental autoimmune encephalomyelitis (EAE) mouse model. Notably, we found that early therapy could significantly reduce the severity of progressive EAE, while treatment initiated at an advanced stage was less efficient. Furthermore, we observed the accumulation of a novel subset of GM-CSF-producing CD11b^+^CD4^+^ T cells in the CNS throughout disease progression. Importantly, early therapeutic anti-VLA-4 mAb treatment suppressed the accumulation of these GM-CSF-producing CD11b^+^CD4^+^ T cells in the CNS along with activated microglia/macrophages populations, and also conferred a protective effect against inflammation-mediated neurodegeneration, including demyelination and axonal loss. Collectively, our data suggest that early treatment with anti-VLA-4 mAb can provide neuroprotection against progressive CNS autoimmune disease by preventing the accumulation of pathogenic GM-CSF-producing CD11b^+^CD4^+^ T cells in the CNS.

## Introduction

Multiple sclerosis is an inflammatory autoimmune disease of the central nervous system (CNS). Throughout the course of MS, invading leukocytes are found to carry out a coordinated attack against myelin and axonal structures through a series of complex effector mechanisms [1].

Early studies which aimed to uncover the mechanisms of leukocyte infiltration across the blood-brain barrier revealed the α_4_β_1_ integrin heterodimer, or very late antigen-4 (VLA-4), to be a critical cellular adhesion molecule in the pathogenesis of experimental autoimmune encephalomyelitis (EAE), the animal model of MS [2], [3]. Preliminary experiments discovered that antibodies against the α-chain of VLA-4 could successfully inhibit pathogenic T cell and monocyte entry into the CNS, resulting in the prevention of EAE [2]–[5]. This outcome eventually led to the development of natalizumab, a humanized anti-VLA-4 monoclonal antibody (mAb), which has been approved for the treatment of RRMS patients and has documented beneficial therapeutic effects [6]. Clinical studies have revealed that gadolinium-enhancing lesions, relapses and axonal damage are reduced in RRMS patients treated with natalizumab [7]–[9]. The accumulation of new cortical lesions and global cortical thinning are also reported to be significantly lower in natalizumab-treated RRMS patients after one and two year follow-up [10], [11]. On the other hand, the effects of natalizumab treatment for primary-progressive MS (PPMS) and secondary-progressive MS (SPMS) remain unclear to date. Likewise, a variety of alternative disease-modifying therapies for these progressive MS types are still lacking, since these therapies have either failed to show promising results or are currently undergoing clinical trials in an attempt to establish a successful progressive MS regimen [12].

Although the effects of inhibiting the VLA-4 adhesion molecule have been studied in previous reports employing a C57BL/6 MOG_35-55_-induced EAE mouse model [13]–[19], these reports have not addressed the therapeutic efficacy of anti-VLA-4 mAb treatment on the progressive stage of the disease. Therefore, we evaluated the efficacy of natalizumab to treat the progressive stage of C57BL/6 MOG_33-55_-induced EAE. Since the expression of CD11b on T cells is crucial for pathogenic T cell development in EAE [20], we also examined the effect of anti-VLA-4 mAb on the development of CD11b^+^CD4^+^ T cells in progressive EAE. Here, we showed that CD11b was up-regulated on CNS-infiltrating pathogenic pro-inflammatory T cells, and that early therapy with anti-VLA-4 mAb could effectively suppress the infiltration of GM-CSF-producing CD11b^+^Th1 cells, including the subsequent accumulation of activated microglia/macrophages. In turn, this led to protection against chronic CNS autoimmune disease progression caused by demyelination and axonal loss. Taken together, these data support the early use of anti-VLA-4 mAb treatment to induce neuroprotection in progressive forms of CNS autoimmune disease by blocking the accumulation of GM-CSF-producing CD11b^+^CD4^+^ T cells in the CNS.

## Materials and Methods

### 1. Induction of EAE and anti-VLA-4 mAb treatment

C57BL/6 mice were purchased from The Jackson Laboratory (Bar Harbor, ME) and housed in a specific pathogen-free facility at the School of Public Health, Rutgers-Robert Wood Johnson Medical School (Piscataway, NJ). EAE was induced by the subcutaneous immunization of 7-week-old C57BL/6 mice with 200 µl emulsions of 200 µg MOG_35-55_ peptide (MEVGWYRSPFSRVVHLYRNGK; Protein and Nucleic Acid Facility, Stanford University, Stanford, CA) in Complete Freund's Adjuvant (4 mg/ml). Additionally, animals received an i.p. injection of 100 µg pertussis toxin (List Biologicals, St. Louis, MO) at Day 0 and 2 to aid disease initiation, and were boosted by MOG_35-55_ (200 µg per mouse)/Incomplete Freund's Adjuvant at Day 7. Then, mice were intraperitoneally administered with anti-VLA-4 mAb (received from Biogen Idec, MA) or IgG1 control (eBioscience, CA) at 5 mg/kg every other day for 10 days. Clinical signs of EAE were assessed according to the following scale: 0: no signs of disease; 1: limp tail; 1.5: paresis of one hindlimb; 2: paresis of both hindlimbs; 2.5: paralysis of one hindlimb; 3.0: paralysis of both hindlimbs; 3.5: paralysis of both hindlimbs and one forelimb paresis; 4: hindlimb paralysis and both forelimb paresis; 5: no mobility/moribund. All animal studies were conducted in accordance with Institutional Animal Care and Use Committee (IACUC) guidelines and were approved by the IACUC through Rutgers-Robert Wood Johnson Medical School Animal Care and Use Committee (Animal Protocol #I10-050-6).

### 2. Flow cytometric analysis

To isolate CNS cellular infiltrates, mice were anaesthetized and perfused intracardially through the left ventricle with 30 ml ice-cold PBS. CNS cell infiltrates were purified with a 30/70% Percoll density gradient after digestion of homogenized brain and spinal cord tissues with Neural Tissue Dissociation Kit (Miltenyi Biotec, Auburn, CA) and passed through 40 micron filter mesh in order to create single cell suspensions. For FACS analysis, 1×10^6^ cells were washed twice with staining buffer (0.1% NaN_3_, 2% FCS in PBS) and stained with antibodies against CD45, CD11b, CD40, TLR-2, MHC class II, CD4, CD3, GM-CSF, IL-17, TNF-α and IFN-γ (eBioscience, CA) according to the manufacturer's protocol. Since CNS-infiltrating encephalitogenic T cells can develop an antigenic specificity to multiple other endogenous epitopes that are presented in the CNS apart from the MOG_35-55_ epitope through the course of EAE [21], the CNS-infiltrates were cultured with plate-bound anti-CD3 mAb (10 µg/ml, eBioscience, CA) plus soluble anti-CD28 mAb (2 µg/ml, BD Pharmingen, CA) in the presence of Brefeldin A (10 µg/ml, Sigma-Aldrich, MO) overnight for intracellular staining. All samples were analyzed on a Cytomics FC500 and Gallios flow cytometer using Kaluza Analysis Software (Beckman Coulter, Inc.). To determine the percentage of positive pro-inflammatory cytokine-producing cells in the CNS, CD11b^+^CD45^+^ and CD4^+^ were sequentially gated, and then the percentage of pro-inflammatory cytokine-producing cells was calculated as ([CD45^+^CD11b^+^ (%)] × [CD4^+^ (%)] × [cytokine ^+^ (%)])/10^4^.

### 3. Immunohistology

Animals were anaesthetized and perfused intracardially with 30 ml ice-cold PBS and 30 ml of 4% paraformaldehyde. Spinal cords were removed and soaked in 30% sucrose/PBS for 3 days. Frozen spinal cord sections (cervical, thoracic, lumber, and sacral regions) were cut at a thickness of 20 µm by cryostat and stained with myelin basic protein (MBP) and neurofilament primary monoclonal antibodies (Abcam) and visualized with fluorescent secondary antibodies for MBP (Goat anti-Rat IgG FITC) and neurofilament (Goat anti-Rat IgG Rhodamine) (Jackson Immunoresearch Laboratories, Inc.). Cell nuclei were counterstained with DAPI for the analysis of total cellular infiltration. Digital images of spinal cord sections were taken with an Axiovert 200 (Zeiss) microscope. Areas of extensive cellular infiltration, demyelination, and axonal loss were manually traced with the AxioVision imaging system onto spinal cord images. Then, the total areas of cellular infiltration, demyelination, and axonal loss in spinal cord sections were calculated by the AxioVision imaging system. The percentage of lesions was calculated as (lesion area/total spinal cord area) ×100.

### 4. Statistical analyses

GraphPad Prism Software (GraphPad Software, Inc., San Diego, CA) was used throughout this study for statistical analyses. EAE clinical scores were evaluated by the non-parametric Mann-Whitney U test or the non-parametric Kruskal-Wallis test followed by a Dunn's post analysis where indicated. P-values for significant suppression of cellular population percentages and cell numbers were calculated by the Student's *t*-test. All p-values ≤0.05 were considered statistically significant.

## Results

### 1. Effect of anti-VLA-4 mAb treatment on the development of progressive EAE

**Figure 1:**
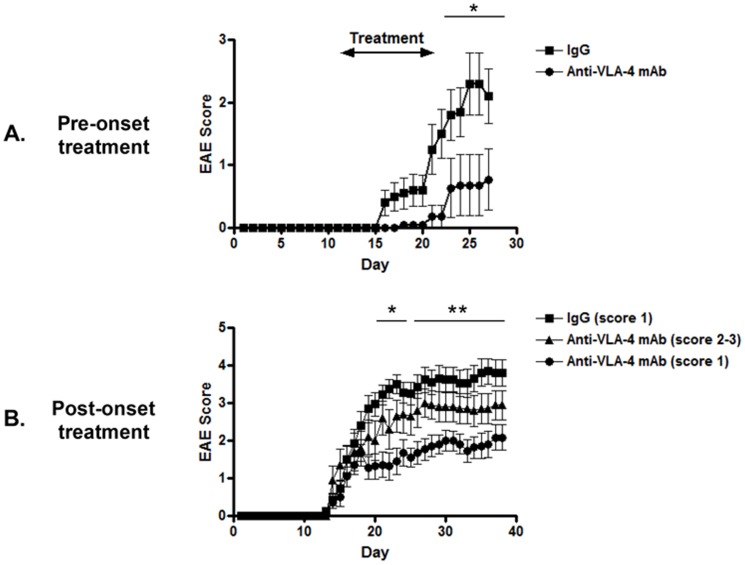
Effect of anti-VLA-4 mAb treatment on the induction of progressive EAE. EAE was induced in C57BL/6 mice by immunization with MOG_35-55_/CFA and injection of pertussis toxin as described in Materials and Methods. (A) Prophylactic treatment; immunized mice were treated (i.p.) with anti-VLA-4 mAb (n = 10) or IgG control (n = 10) at 5 mg/kg every other day for 10 days at Day 13 post-immunization before disease onset. *; p-value <0.05 by Mann-Whitney U test. (B) Post-onset treatment; When disease score reached to grade 1 or grade 2–3, the EAE mice were treated with anti-VLA-4 mAb every other day for 10 days. As a control, mice with disease grade 1 were treated with IgG for 10 days. Data shown are the mean clinical score (+/− SEM) of 10-16 mice in each group from three experiments. *; p-value <0.05, **; p-value <0.01for the comparison between IgG (score 1) and anti-VLA-4 mAb (score 1) by Kruskal-Wallis test.

MOG_35-55_-induced EAE on the C57BL/6 mice is a well-established animal model of progressive CNS autoimmune disease, whereby inflammation, demyelination, and axonal loss steadily accrue and are thought to lead to clinical deficit [22]. Therefore, we employed this animal model in our study to assess whether anti-VLA-4 mAb treatment could confer protection against progressive EAE development. To confirm a similar result to alternate models of EAE treated with anti-VLA-4 mAb or antagonist [13], [15], [23], we first examined whether prophylactic (pre-onset) treatment with anti-VLA-4 mAb could prevent the induction of EAE. Since the onset of EAE was determined to ensue around Day 16 post-immunization, we introduced our prophylactic regimen of 5 mg/kg anti-VLA-4 mAb or IgG control on Day 13 post-immunization and continued this regimen every other day for 10 days. In agreement with previous data, prophylactic treatment with anti-VLA-4 mAb could significantly suppress the induction of progressive EAE (Fig. 1A).

Next, to evaluate the therapeutic efficacy of anti-VLA-4 mAb in progressive EAE, we examined whether post-onset anti-VLA-4 mAb treatment for early and advanced EAE could mitigate the severity of EAE. In order to properly treat the mice at these respective disease stages, mice that exhibited early clinical signs of EAE (disease grade 1) were selected for either early anti-VLA-4 mAb or IgG control treatment every other day for 10 days, while mice that showed advanced signs of EAE (disease grade 2–3) were given late anti-VLA-4 mAb treatment every other day for 10 days. Markedly, we found that early anti-VLA-4 mAb treatment could significantly suppress disease progression when administered during early clinical signs of EAE (Fig. 1B). While early-stage EAE mice developed disease progression after stopping treatment, their disease severity was prominently less than that of IgG-treated mice at an early stage of EAE (Fig. 1B). However, in an advanced stage of EAE, we were unable to observe a significant therapeutic effect of anti-VLA-4 mAb treatment (Fig. 1B). Therefore, these data indicate that anti-VLA-4 mAb treatment is able to suppress progressive EAE more efficiently when initiated at an early stage of disease.

### 2. Effect of early anti-VLA-4 mAb treatment on pathogenic CNS-infiltrating GM-CSF^+^CD4^+^ T cells

**Figure 2:**
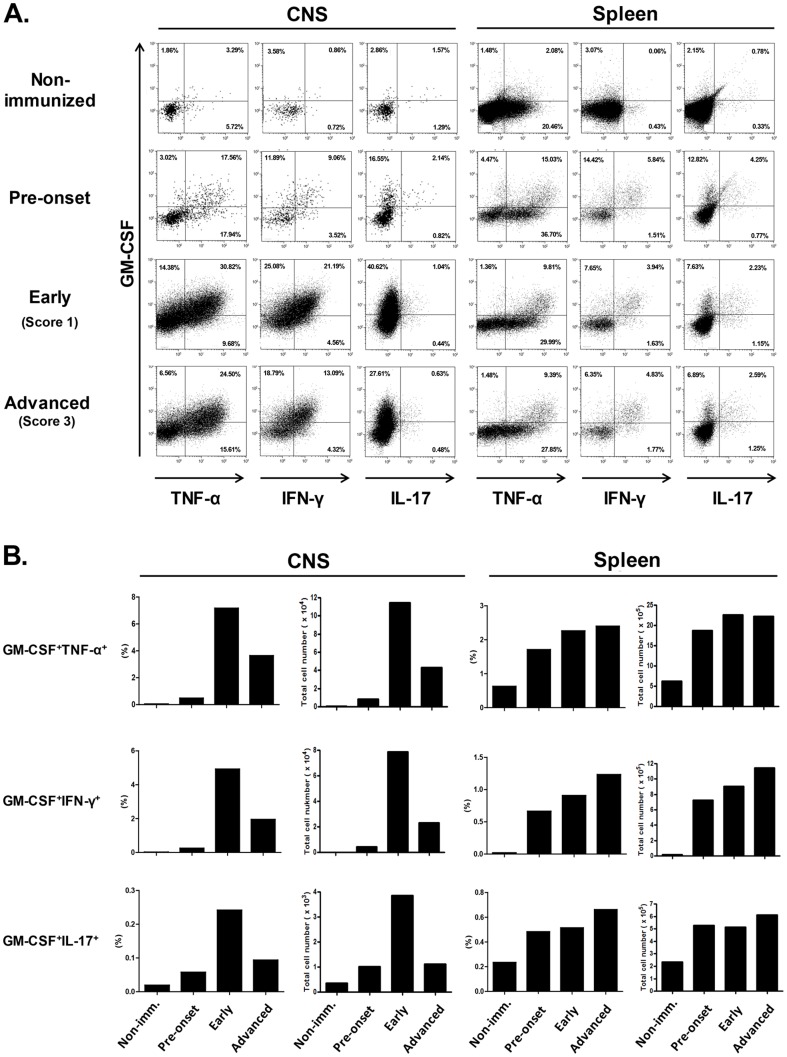
GM-CSF^+^ Th1 and Th17 cell development in the spleen and infiltration into the CNS. (A) The CNS (brain and spinal cord) and spleen were harvested from non-immunized mice and mice with prophylactic, early (disease grade 1), and advanced (disease grade 3) stages of EAE. The CNS infiltration of pro-inflammatory T cells was analyzed by measuring the production of GM-CSF, TNF-α, IFN-γ, and IL-17 in response to stimulation with anti-CD3/CD28 mAbs. CD11b^-^CD45^+^ CD4^+^ leukocytes were gated for the analysis of cytokine production. (B) Percentages and total cell number of GM-CSF^+^IL-17^+^, GM-CSF^+^IFN-γ^+^, and GM-CSF^+^TNF-α^+^ T cells in the CNS and spleen. The percentage of infiltrated cells was calculated as described in the Materials and Methods. The representative data were confirmed in two independent experiments.

We next aimed to assess the therapeutic effect of anti-VLA-4 mAb treatment on the infiltration of pathogenic pro-inflammatory T cells into the CNS. In order to properly evaluate the therapeutic effect, we first proceeded to characterize the pro-inflammatory phenotypes and infiltration patterns of the invading cells during each stage of progressive EAE development. Considering that granulocyte macrophage colony-stimulating factor (GM-CSF) cytokine production is crucial for the encephalitogenic potential of infiltrating T cells and the onset of EAE [24]–[26], we specifically analyzed the CNS-infiltration of GM-CSF^+^ T helper (Th)1 and Th17 cell populations. As shown in Fig. 2A, both GM-CSF^+^Th1 and GM-CSF^+^Th17 cells developed in the spleen of immunized mice during the pre-onset period and further expanded after the disease onset (Fig. 2A). In the CNS, the infiltration of GM-CSF^+^Th1 cells was more frequently observed compared to that of GM-CSF^+^Th17 cells (Fig. 2A). Moreover, populations of CNS-infiltrating GM-CSF^+^Th1 and GM-CSF^+^Th17 cells were only marginally increased at the pre-onset stage compared to non-immunized mice; however, at an early stage of EAE progression, CNS-infiltrating GM-CSF^+^Th1 and GM-CSF^+^Th17 cells were greatly elevated and thus represented the peak level of pathogenic T cell infiltration (Fig. 2B). By an advanced stage of EAE, CNS-infiltrating GM-CSF^+^Th1 and GM-CSF^+^Th17 cells had decreased relative to the peak early stage albeit their continued development in the spleen (Fig. 2B).

**Figure 3:**
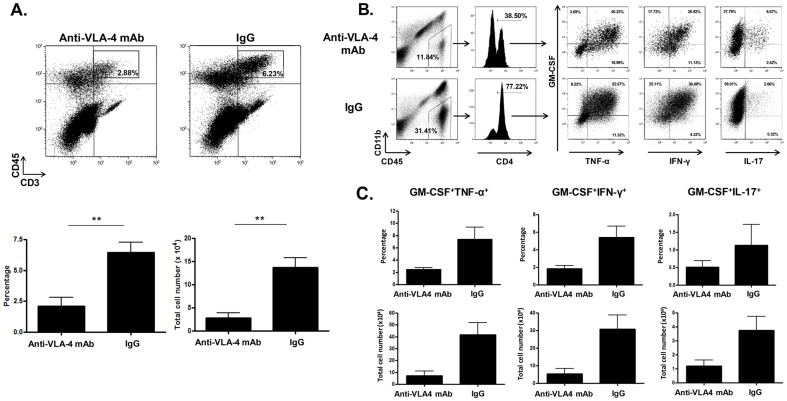
Effect of early therapeutic anti-VLA-4 mAb on pathogenic T cell infiltration into the CNS. (A) Mice were sacrificed one day after last treatment and mononuclear cells isolated from the CNS (brain and spinal cord) of anti-VLA-4 mAb-treated (n = 4) and IgG-treated (n = 4) mice were stained by anti-CD45 and -CD3 mAbs. CNS-infiltrated T cells (CD45^+^CD3^+^) were analyzed by flow cytometry. **; p-value <0.01. (B) Production of pro-inflammatory cytokine by the CNS-infiltrated CD4 T cells in anti-VLA-4 mAb- and IgG-treated mice. CD11b^+^CD45^+^ and CD4^+^ were sequentially gated, and then production of pro-inflammatory cytokines in response to anti-CD3/CD8 mAb in the CD11b^+^CD45^+^CD4^+^ cells was examined. (C) Effect of anti-VLA-4 mAb treatment on CNS-infiltration of GM-CSF^+^Th1 and GM-CSF^+^Th17 cells. CNS-infiltration of GM-CSF^+^TNF-α^+^, GM-CSF^+^IFN-γ^+^, and GM-CSF^+^IL-17^+^ CD4^+^ T cells in anti-VLA-4 mAb (n = 3) and IgG (n = 4)-treated mice were calculated as described in Materials and Methods. Data represent mean +/− SEM. Results shown are representative from three experiments.

Since early anti-VLA-4 mAb treatment significantly reduced the severity of EAE in comparison to advanced-stage treatment (Fig. 1B), we then assessed whether this treatment could suppress the peak of pathogenic T cell infiltration into the CNS. Interestingly, the overall percentage of CNS-infiltrating CD3^+^ T cells was significantly reduced by anti-VLA-4 mAb treatment administered at an early stage of EAE (Fig. 3A). Furthermore, to specifically examine the effect of early anti-VLA-4 mAb treatment on the CNS-infiltration of GM-CSF^+^Th1 and GM-CSF^+^Th17 cells, the frequency of these pathogenic CD4^+^ T cells was calculated by the sequential gating of CD45^+^CD11b^l^°CD4^+^ cells in the CNS of non-treated and treated EAE mice (Fig. 3B and 3C). As shown in Fig. 3B, early anti-VLA-4 mAb treatment suppressed the infiltration of both GM-CSF^+^Th1 and GM-CSF^+^Th17 cells; however, the suppressive efficiency of GM-CSF^+^Th1 cells was slightly more effective compared to GM-CSF^+^Th17 cells (Fig. 3B). Thus, our data suggests that early anti-VLA-4 mAb treatment can prevent the on-going infiltration of the CNS by pathogenic CD4^+^ T cells to help block the disease progression of EAE.

### 3. Effect of early anti-VLA-4 mAb treatment on the development of CD11b^+^ CD4^+^ T cells in the CNS

**Figure 4:**
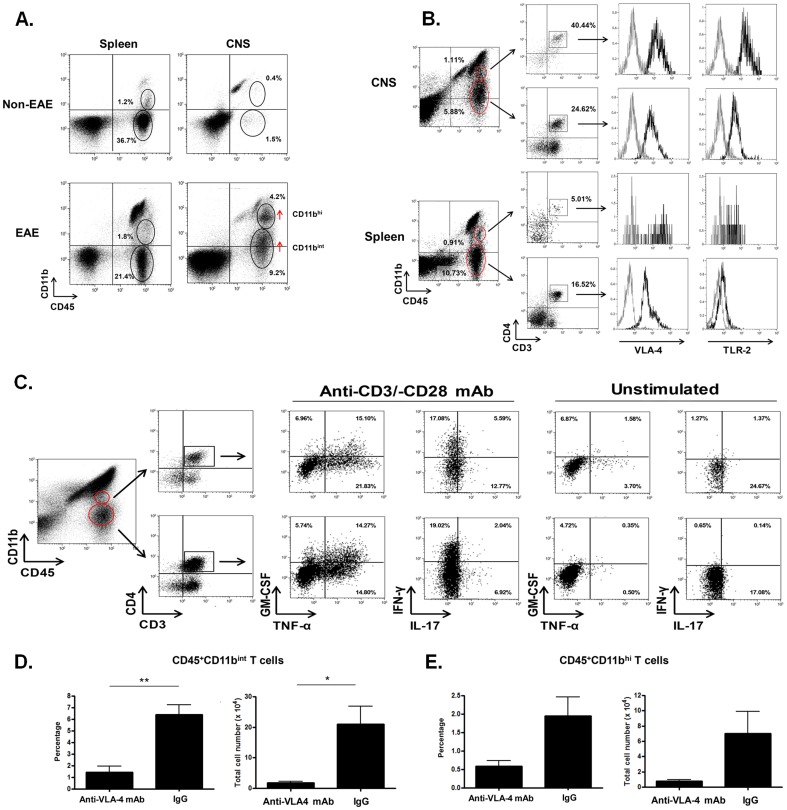
Effect of early therapeutic anti-VLA-4 mAb on the CNS-infiltration and/or development of CD45^+^CD11b^+^ T cells. (A) Up-regulation of CD11b on CNS-infiltrated CD45^+^ cells compared to splenic CD45^+^ cells in EAE mice (disease grade 3). (B) Expression of CD3, CD4, VLA-4 and TLR-2 on CD45^+^CD11b^int^ and CD45^+^CD11b^hi^ cells in the CNS of EAE mice (disease grade 3.5). As a negative control staining, isotype-matched IgG was used for the staining (gray histogram). (C) Production of GM-CSF, TNF-α, IFN-γ, and IL-7 in CD45^+^CD11b^int^ and CD45^+^CD11b^hi^ CD4^+^ T cells. CD11b^+^CD45^+^ and CD4^+^ were sequentially gated, and the production of pro-inflammatory cytokines in CD11b^+^CD45^+^CD4^+^ cells in the presence or absence of anti-CD3/-CD28 mAbs was examined. (D,E) Suppression of CNS infiltration/development of (D) CD45^+^CD11b^int^ T cells and (E) CD45^+^CD11b^hi^ T cells by 7 day-early therapeutic treatment with anti-VLA-4 mAb (n = 3) and IgG (n = 4). **; p-value <0.01. Data represent mean +/− SEM. Results shown are representative from three experiments.

It has been demonstrated that pathogenic T cells require CD11b expression to mediate the development of EAE [20]. Therefore, we assessed whether the up-regulation of CD11b on CNS-infiltrating T cells was observed during the progressive stage of EAE. To do so, we first examined the expression of CD11b on all CNS-infiltrating leukocytes (CD45^+^ cells) in EAE mice. Flow cytometric analysis led to our detection of a minor population of splenic CD45^+^CD11b^+^ cells (1.2% in non-EAE mice and 1.8% in EAE mice) expressing CD11b at an intermediate level (CD11b^int^) in addition to CD45^+^CD11b^+^ macrophage populations present in the spleen (Fig. 4A). In the CNS of EAE mice, however, the expression of CD11b was up-regulated on CD45^+^ cells and two distinct cellular populations became evident (CD45^+^CD11b^int^ and CD45^+^CD11b^hi^) apart from resting microglia and activated microglia/macrophage populations which we address later in this study (Fig. 4A). We then examined the expression of the CD3 and CD4 T cell markers on CD45^+^CD11b^int^ and CD45^+^CD11b^hi^ cell populations. Interestingly, 40.44% and 24.62% of CD45^+^CD11b^hi^ and CD45^+^CD11b^int^ cell populations, respectively, expressed both CD3 and CD4 (Fig. 4B), which indicates that CD11b^+^CD4^+^ T cells accumulate in the CNS of progressive EAE mice. Furthermore, both populations expressed VLA-4 and Toll-like receptor (TLR)-2 (Fig. 4B), and produced pro-inflammatory cytokines, including GM-CSF, TNF-α, IFN-γ, and IL-17 in response to anti-CD3/-CD28 stimulation (Fig. 4C). Notably, early anti-VLA-4 mAb treatment significantly suppressed the accumulation of CD45^+^CD11b^int^ T cells in the CNS (Fig. 4D), while the suppression of the CD45^+^CD11b^hi^ T cell population followed a similar trend (Fig. 4E). In turn, these data indicate that early anti-VLA-4 mAb treatment can suppress the CNS-infiltration and/or development of GM-CSF-producing CD11b^+^TLR-2^+^CD4^+^ T cells.

### 4. Effect of early anti-VLA-4 mAb treatment on the accumulation of activated microglia and macrophages in the CNS

**Figure 5:**
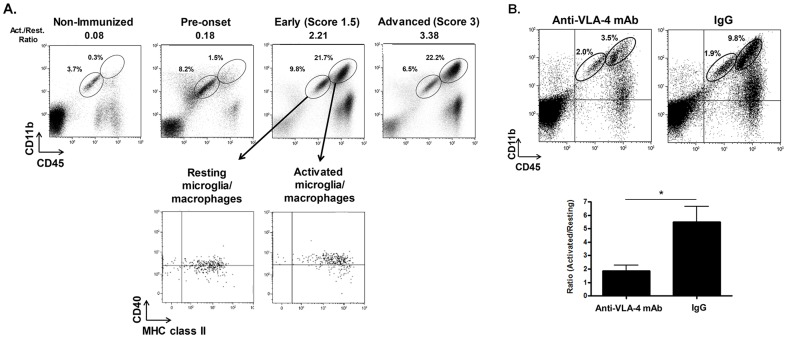
Effect of early therapeutic anti-VLA-4 mAb on microglia/macrophage activation in the CNS. (A) The conversion of resting microglia/macrophages into activated microglia/macrophages is associated with disease progression. Phenotypes of resting and activated microglia/macrophages are CD45^low^CD11b^+^ and CD45^hi^CD11b^+^, respectively. Expression levels of MHC class II and CD40 on the resting and activated microglia/macrophages are shown. Resting microglia/macrophages: CD40 (Mean Fluorescence Intensity (MFI): 2.4) and MHC class II (MFI: 7.33). Activated microglia/macrophages: CD40 (MFI: 4.24) and MHC class II (MFI: 40.5). The percentage ratio between activated and resting macrophages (Act./Rest. Ratio) increased with disease progression. (B) Early therapeutic anti-VLA-4 mAb treatment suppresses the activation of microglia/macrophages. Mice were sacrificed one day after the last treatment and the conversion of resting microglia/macrophages into activated microglia/macrophages was examined by measuring the percentage ratio between activated vs. resting microglia/macrophages in anti-VLA-4 mAb-treated (n = 5) compared to IgG-treated (n = 6) mice. Data represent mean +/− SEM. *; p-value <0.05.

The activation of microglia/macrophages is a key step in mediating EAE pathology, as these cells are frequently found to co-localize with encephalitogenic T cells in inflamed tissues [27]. The initial activation of microglia is characterized by a shift in the expression of CD45 from a low level in steady-state resting microglial populations (CD45^l^°CD11b^+^) to a high level in activated microglial populations (CD45^hi^CD11b^+^) [28]. Moreover, since the phenotype of activated infiltrating macrophages resembles that of activated microglia, the CD45^hi^CD11b^+^ cell population consists of both activated microglia and peripheral macrophages. As demonstrated in Fig. 5A, CD45^l^°CD11b^+^ resting microglia cells are the primary population at steady-state in the CNS of non-immunized mice. In mice developing EAE, however, the predominant population in the inflamed CNS becomes activated microglia/macrophages of the CD45^hi^CD11b^+^ phenotype (Fig. 5A). Since activated microglia/macrophages also express elevated levels of major histocompatibility complex (MHC) class II and CD40 in the CNS [28], immunostaining for the up-regulation MHC class II and CD40 expression was performed and subsequently confirmed (Fig. 5A). Thus, the activation of CNS microglia and macrophages can be monitored by the percentage ratio between activated and resting microglia/macrophage populations, as this ratio is found to increase with disease progression in EAE mice (Fig. 5A).

To then evaluate the ability of early anti-VLA-4 mAb treatment to suppress microglial and macrophage activation in the CNS throughout the course of progressive EAE, we assessed whether the administration of early anti-VLA-4 mAb could reduce the ratio of activated CD45^hi^CD11b^+^ microglia/macrophage populations versus resting CD45^l^°CD11b^+^ microglial populations. As shown in Fig. 5B, the accumulation of activated CD45^hi^CD11b^+^ microglia/macrophages in the CNS was significantly suppressed by early anti-VLA-4 mAb treatment. Therefore, this data reveals that early anti-VLA-4 mAb therapy can prevent the activation of microglia and/or the infiltration of the CNS by activated peripheral macrophages after the onset of EAE.

### 5. Effect of early anti-VLA-4 mAb treatment on neurodegeneration

**Figure 6:**
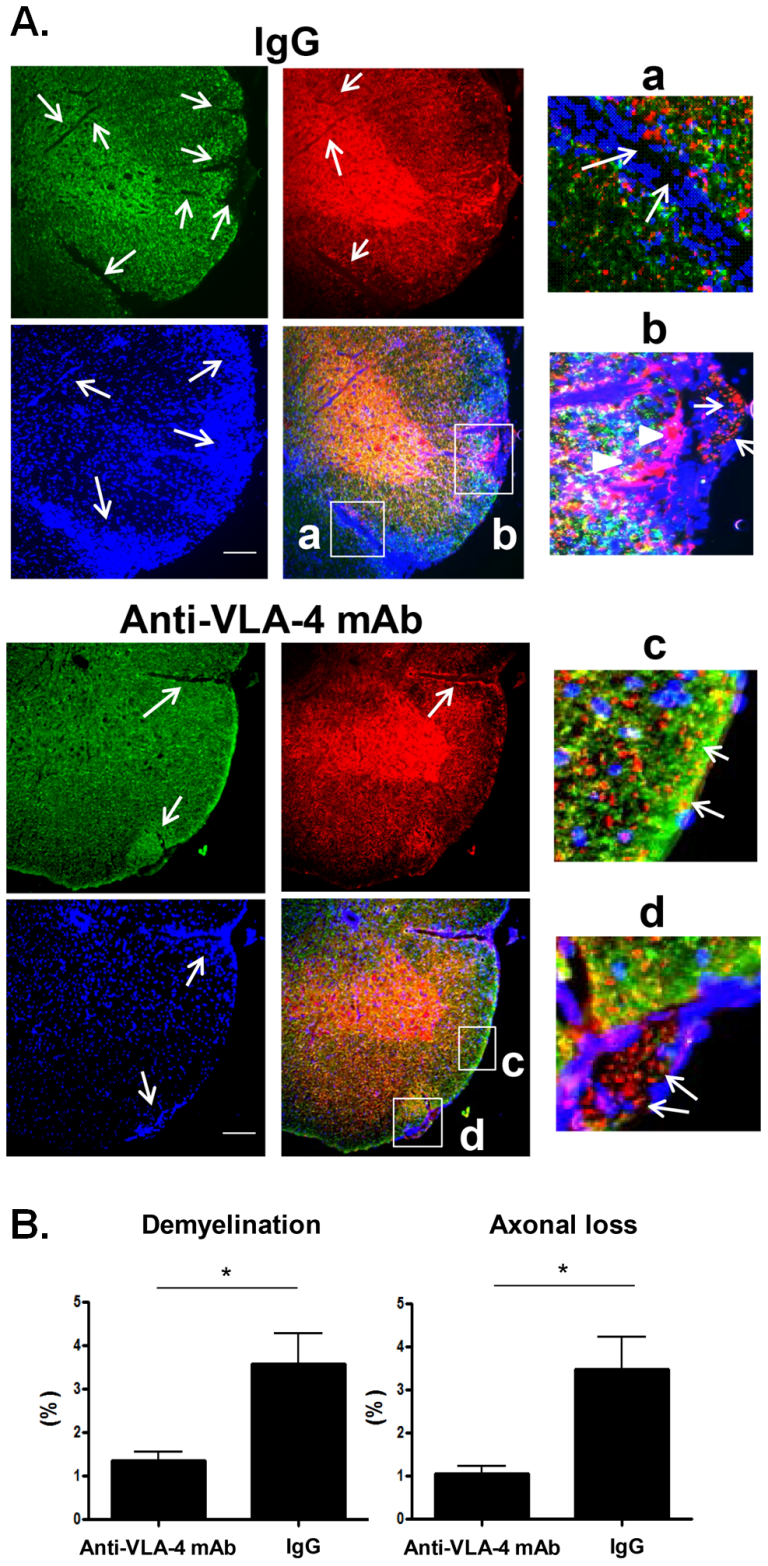
Effect of anti-VLA-4 mAb treatment on CNS cellular infiltration, demyelination and axonal loss. (A) Mice were sacrificed 40-46 days after immunization and cell infiltration, demyelination, and axonal loss were examined by immunohistology. Four spinal cord sections (cervical, thoracic, lumber, and sacral regions) were prepared from early therapeutic anti-VLA-4 mAb- and IgG-treated EAE mice. Demyelination and axonal loss were examined by staining with anti-myelin basic protein (MBP) and anti-neurofilament (NF) Abs, respectively. Cell nuclei were counterstained with DAPI and accumulation of cell infiltrates was detected by clusters of DAPI-positive cells. (a) An area showing both demyelination and axonal loss induced by infiltrated cells is shown by arrows. (b) Swelling axons (arrow head) and demyelinated axons (arrow) are shown. (c) Myelinated and (d) demyelination axons are shown. Staining of the lumber spinal cord section is shown. Scale: 100 µm. (B) Anti-VLA-4 mAb treatment suppresses demyelination and axonal loss in the CNS (brain and spinal cord). Demyelination and axonal loss were examined by measuring the area stained by MBP Ab and NF Ab, respectively, and the percentage of each area was calculated as described in the Materials and Methods. Anti-VLA-4 mAb-treated mice; n = 5 and IgG-treated mice; n = 4. Data represent mean +/− SEM. *; p-value <0.05

Since activated microglia and macrophages polarized toward an M1 phenotype produce pro-inflammatory cytokines and carry out effector mechanisms that induce demyelination and axonal loss during CNS neuroinflammation [29], [30], we sought to determine the effects of early anti-VLA-4 mAb treatment on both demyelination and axonal loss. During EAE progression, we observed that demyelination was predominantly detected in sites where large areas of cellular infiltrates were localized (Fig. 6A). Axonal loss was also commonly found to accompany demyelination (Fig. 6A–a), although there was evidence of primary demyelination without axonal loss (Fig. 6A–b and -d) and axonal swelling (Fig. 6A–b) in some cases. On the other hand, axonal loss without demyelination was rarely detected.

Thereafter, we assessed whether early anti-VLA-4 mAb treatment could confer protection against demyelination and axonal loss in early-stage EAE mice between 40–46 days post-immunization. Remarkably, demyelination and axonal loss were significantly suppressed by early anti-VLA-4 mAb treatment compared to IgG treatment after the onset of disease (Fig. 6B). Collectively, these results indicate that anti-VLA-4 mAb treatment is beneficial for the suppression of inflammation-mediated neurodegeneration when administered early in the course of EAE.

## Discussion

Several earlier reports have demonstrated the prophylactic efficacy of anti-VLA-4 mAb or antagonist treatment in either PLP_139-151_-induced relapsing EAE in SJL mice or bovine MBP-induced chronic relapsing EAE in the SJL/J mice [13], [15], [23]. On the contrary, anti-VLA-4 mAb or antagonist treatment was ineffective after the onset of disease in these studies [13], [15], [23]. In agreement with these aforementioned reports, our present study demonstrated a suppressive effect of prophylactic anti-VLA-4 mAb treatment for progressive EAE (Fig. 1A), whereas treatment at an advanced stage of progressive EAE did not confer disease amelioration (Fig. 1B). Interestingly, however, we observed a beneficial response to early anti-VLA-4 mAb treatment administered shortly after the onset of progressive EAE (Fig. 1B). Thus, our data suggests that the timing of anti-VLA-4 mAb treatment is a crucial factor to effectively treat progressive CNS autoimmune diseases. However, since disease progression occurred after 10 days of anti-VLA-4 mAb treatment, a longer duration of treatment may be required to gain a greater therapeutic outcome. A similar observation was also reported during the early treatment of progressive EAE with anti-MAdCAM-1 mAb. Although MAdCAM-1 is a ligand for the gut-homing integrin, α_4_β_7_, early treatment with anti-MAdCAM-1 mAb was able to suppress disease progression in MOG_35-55_-induced EAE [31].

Upon further evaluation of early anti-VLA-4 mAb treatment in progressive EAE, we showed that pathogenic GM-CSF^+^Th1 cells highly infiltrated the CNS at an early stage of disease (Fig. 2A and 2B), and that early anti-VLA-4 mAb treatment could suppress the accumulation of GM-CSF^+^Th1 cells during the peak of CNS-infiltration (Fig. 3B and 3C). Although the CNS-infiltration of GM-CSF^+^Th17 cells was less frequently observed compared to that of GM-CSF^+^Th1 cells, early anti-VLA-4 mAb was able to block the infiltration of GM-CSF^+^ Th17 cells into the CNS; however, this blockage was slightly less effective for GM-CSF^+^Th17 cells (Fig. 3C). More recently, a study indicated that VLA-4 is the primary integrin for Th1 cell infiltration into both the brain and spinal cord, whereas the LFA-1 and VLA-4 integrins are required for Th17 cell infiltration into the brain and spinal cord, respectively [17]. In this case, it is possible that anti-VLA-4 mAb treatment might be less effective to suppress the CNS-infiltration of GM-CSF^+^Th17 cells compared to that of GM-CSF^+^Th1 cells because CNS infiltrates were isolated from the combined tissues of the brain and spinal cord in this study. Although T cells that infiltrate the CNS tend to undergo apoptosis in the CNS [32], [33], the accumulation of pathogenic T cells by their continuous infiltration may induce the chronic inflammation necessary for disease progression toward severe neurodegeneration. Therefore, a threshold level of CNS-infiltrating GM-CSF^+^ pathogenic T cells may exist that can promote enough neuroinflammation in the CNS to trigger neurodegeneration. Considering we found the CNS-infiltration of GM-CSF^+^Th1 and GM-CSF^+^Th17 cells to be highest at an early stage of disease progression, it is possible that early therapy may be able to prevent the further accumulation of pathogenic T cells in the CNS in order to suppress disease progression.

Given that infiltrating pathogenic GM-CSF-producing CD4^+^ T cells contribute to the activation of microglia/macrophages, it is likely that early therapeutic anti-VLA-4 mAb treatment is able to suppress the activation of microglia/macrophages in the CNS as a direct consequence of blocking GM-CSF^+^CD4^+^ T cell infiltration (Fig. 5B). GM-CSF produced by infiltrating pathogenic T cells activates microglia/macrophages to induce a neuroinflammatory cascade in the pathogenesis of EAE. Following their activation, microglia/macrophages produce various pro-inflammatory mediators that provoke detrimental CNS mechanisms, including demyelination, hypomyelination, and cell death [30], [34]. Moreover, the infiltration of the CNS by peripheral macrophages has also been shown to be directly associated with the development of EAE [35], [36]. Throughout the course of EAE, infiltrating macrophage populations comprise the majority of CD11b^+^ cell populations in the CNS during the onset and peak of disease, and are found to express higher levels of activation markers such as MHC class II, CD86, CD40 and CD11c [27], [28]. In addition, macrophages have been demonstrated to use the VLA-4 adhesion molecule as a primary integrin for CNS infiltration [37], [38]. Therefore, early therapeutic anti-VLA-4 mAb treatment may also prevent the infiltration of activated macrophages into the CNS to help suppress disease progression.

Our observations which also highlighted the appearance of pro-inflammatory CD4^+^ T cells that up-regulated CD11b and VLA-4 expression in the CNS during progressive EAE suggest the involvement of these CD11b^+^CD4^+^ T cells in the progression of EAE (Fig. 4A-C). Not to mention, we also observed the appearance of a population of CD11b^+^CD3^+^CD4^−^ cells in the CNS (Fig. 4B) found to contain CD8^+^ T cells (data not shown). Interestingly, the expression of CD11b on T cells is necessary to induce EAE [20]; however, pathogenic role of CD11b in EAE progression is still unknown. Since CD11b is not required for pathogenic T cell infiltration into the CNS [20], it is possible that CD11b may be more involved in the full differentiation of pro-inflammatory effector T cells that contribute to neuroinflammation. In humans, CD11b^+^ T cells represent a minor population that can be detected in the peripheral blood, spleen, and liver [39]. In addition, the expression of the IFN-γ, IL-2, and IL-4 genes are up-regulated in CD11b^+^ T cells and their numbers are correlated with an increase in age [39], [40]. Furthermore, CD11b^+^ T cells are found to possess a fully-activated effector phenotype and have been detected in scenarios of chronic antigenic stimulation, such as in persistent bacterial or viral infections and neoadjuvant cancer therapy [41]-[46]. Our analysis of early anti-VLA-4 mAb treatment on pathogenic T cells revealed that treatment suppressed the infiltration and/or expansion of CD45^+^CD11b^int^ and CD45^+^CD11b^hi^ CD4^+^T cells in the CNS of progressive EAE mice (Fig. 4A and D-E). At this point, it is unknown whether CD45^+^CD11b^int^ CD4^+^ T cells represent a distinct T cell population apart from CD45^+^CD11b^hi^ CD4^+^ T cells, or whether they represent infiltrating T cell populations capable of converting into CD45^+^CD11b^hi^ CD4^+^T cells inside the CNS. We also detected the expression of TLR-2 on CD11b^+^CD4^+^ T cell populations in the CNS of progressive EAE mice. The expression of TLR-2 is already known to be induced on activated CD4^+^ T cells and is constitutively expressed on effector memory CD4^+^ T cell populations [47], in addition to effector cells derived from patients with persistent bacterial infections and tuberculosis [45], [48]. In contrast to innate immune cells, TLR-2 can serve as a co-stimulatory signal for T cells to promote the development of both Th1 and Th17 populations [49], [50]. Moreover, studies suggest a role for TLR-2 in MS pathogenesis, since T cells lacking TLR-2 can only induce mild clinical EAE associated with lower CD4^+^ and CD11b^+^ cell infiltration of the CNS [50], [51]. Hence, the CD11b^+^TLR-2^+^CD4^+^ T cells detected within the CNS of progressive EAE mice in our study may represent a group of pathogenic effector T cells which are recruited from the peripheral blood and later up-regulate CD11b and TLR-2 upon full activation in the CNS. Thus, it is possible that MOG_35–55_-specific pathogenic CD4^+^ T cells first infiltrate the CNS after undergoing activation in the periphery. Then, these infiltrated CD4^+^ T cells may become further activated by cognate antigens in the CNS, which prompts their differentiation into CD11b^+^TLR-2^+^CD4^+^ T cells. Markedly, we also detected the presence of CD11b^+^TLR-2^+^CD4^+^ T cells in the CNS of MBP-specific TCR/HLA-DR2a Tg mice upon their development of spontaneous EAE [52] (data not shown). This indicates that the development of CD11b^+^TLR-2^+^CD4^+^ T cells is not unique to inducible active EAE, and therefore, this cellular population might be involved in the development of spontaneous EAE as well. Since the accumulation of CD11b^+^TLR-2^+^CD4^+^ T cells in the CNS was associated with increasing neurodegeneration, future experiments are required to assess the pathogenic role of CD11b^+^TLR-2^+^CD4^+^ T cells in regards to their possible neurodegenerative effector functions.

Our data suggest that the accumulation of GM-CSF-producing CD11b^+^CD4^+^ T cells is associated with disease progression in EAE, and that early anti-VLA-4 mAb therapy can prevent the accumulation of GM-CSF^+^CD11b^+^CD4^+^ T cells in the CNS by administration early in the course of disease progression. The suppression of pathogenic GM-CSF-producing T cell infiltration and subsequent microglia/macrophage activation by early anti-VLA-4 mAb treatment further confirms the beneficial effect of this drug to promote neuroprotection against inflammation-mediated neurodegeneration in progressive CNS autoimmunity. This suggests that natalizumab may be an effective therapy for patients with progressive forms of MS, and thus, may aid to prevent disease progression and severe neurodegeneration as long as treatment is administered early after the disease diagnosis.
